# Deimplementing Untested Practices in Homecare Services: A Preobservational-Postobservational Design

**DOI:** 10.1155/2019/5638939

**Published:** 2019-03-19

**Authors:** Manon Guay, Mélanie Ruest, Damien Contandriopoulos

**Affiliations:** ^1^School of Rehabilitation, Faculty of Medicine and Health Sciences, Université de Sherbrooke, J1H 5N4, Canada; ^2^Research Centre on Aging, Centre Intégré Universitaire de Santé et de Services Sociaux de l'Estrie-Centre Hospitalier Universitaire de Sherbrooke, J1H 4C4, Canada; ^3^Research Programs in Health Sciences, Faculty of Medicine and Health Sciences, Université de Sherbrooke, J1H 5N4, Canada; ^4^School of Nursing, University of Victoria, V8W 2Y2, Canada

## Abstract

**Introduction:**

With community-dwelling elders waiting to adapt their bathroom, Health and Social Services Centers in Quebec (Canada) combined human resources through cross-skilling within interdisciplinary teams. To this end, occupational therapists implemented in-house “tools” to support nonoccupational therapists in selecting bathing equipment. However, unknown psychometric properties of those in-house “tools” cast doubt on the quality of service provided to elders. Little is also known about the best processes to use to support the deimplementation of such nonevidence-based practices. This study presents the effect of a knowledge transfer and exchange intervention designed to deimplement in-house “tools” and replace them with an evidence-based tool (Algo).

**Methods:**

Censuses were conducted with the 94 Health and Social Services Centers of Quebec providing homecare services, before and after the knowledge transfer and exchange intervention (2009-2013). In 2013, the deimplementation of in-house “tools” and their replacement by Algo were measured with Knott and Wildavsky's levels of utilization.

**Results:**

Cross-skilling within interdisciplinary teams increased between censuses (87% to 98%), as did use of in-house “tools” (67% to 81%). Algo's uptake started during the knowledge transfer and exchange process as 25 Health and Social Services Centers achieved the first level of utilization. Nonetheless, no Health and Social Services Center deimplemented the in-house “tools” to use Algo.

**Conclusion:**

The knowledge transfer and exchange process led to the development of a scientifically sound clinical tool (Algo) and challenged the status quo in clinical settings regarding the use of nonevidence-based practices. However, the deimplementation of in-use practices has not yet been observed. This study highlights the need to act proactively on the deimplementation and implementation processes.

## 1. Introduction

Healthcare services for elders have been internationally reoriented from acute care to the elders' own home over the last decades [1]. With the increased wait time in homecare services [2], homecare stakeholders want to intervene quickly to prevent injury and help elders remain independent and stay at home [3]. Among the work particularities in homecare services, team member skill mix is an organizational method used to optimize the provision of services [4]. Skill mix can be defined as a flexible way to combine human resources through extended roles and cross-skilling within an interdisciplinary team [5]. Among the team members, the occupational therapists make it possible for people to perform occupations fostering health and well-being. Referrals to occupational therapists often relate to bathing difficulties, the most problematic activity of daily living associated with aging [6]. According to the Quebec (Canada) regulatory board of occupational therapists, overlapping of roles is acceptable, provided that nonoccupational therapists rely on “tools.” For example, nonoccupational therapists should use a clinical decision algorithm to select bathing equipment (e.g., grab bars, bath seats) for home-dwelling elders facing challenges while performing personal hygiene tasks [7, 8].

A census conducted with Quebec homecare services in 2009 revealed that at least 52 different in-house “tools” were implemented by clinicians to meet the board requirements [9]. These local initiatives add confusion to how skill mix should be implemented in occupational therapy [9] and limit worker mobility [10]. Moreover, the unknown psychometric properties and evidence base of those in-house “tools” shed doubt on the quality of the services provided to elders [9]. To enhance the efficiency of processes and evidence-based practice in homecare occupational therapy, a knowledge transfer and exchange (KTE) intervention was designed through the development of a common clinical decision algorithm, named “Algo” [11]. Algo is a decisional map illustrating the steps to follow to select bathing equipment for “straightforward” cases, i.e., cases representing clients with standard morphological and environmental characteristics, with predictable occupational performance during bath transfer [11, 12]. The clinical algorithm is comprised of 4 different sections. The first 2 sections contain dichotomous questions related to the client, the environment (i.e., bathroom) and the occupation (i.e., hygiene care). The first section allows to identify if the client represents a straightforward case. If this is the case, Section 2 can be completed by the nonoccupational therapist, at the client's home, in order to identify the recommendations for bathing equipment. Nonmandatory Sections 3 and 4 allow, respectively, to give general recommendations to the client and visual specifications to the occupational therapist if a discussion is necessary following the identification process at home [13]. Since our goal was to replace the current practice (i.e., in-house “tools”) for selecting bathing equipment with an innovation (i.e., Algo)—a process described as “substitution” by van Bodegom-Vos et al. [14]—implementation must be preceded by deimplementation.

Although there is a growing body of knowledge in literature about implementation science in occupational therapy [15–18], there is much less information available on the deimplementation process of untested practices [14]. For decades, the collaboration between knowledge producers and users has been recognized as a strong facilitation characteristic in the implementation process [19], leading to interactive models of research utilization. Despite the differences between deimplementation and implementation processes, [14] the interactive nature of research appears to influence both of them, the stakeholder engagement being highlighted to specifically guide the deadoption of low-value practices [20]. Little is however known about the clinical impact of using a KTE intervention on the deimplementation process.

To optimize the adoption of best practices as well as the deimplementation of untested practices, the development and implementation of Algo was therefore based on an interactive model of research utilization [19]. The underlying assumption for the study was that the KTE intervention could challenge conditions under which previously legitimated organizational actions hamper the deinstitutionalization of nonevidence-based practices, which is an important aspect of deimplementation [21]. The purpose of this paper is to describe this KTE intervention and its effects on the deimplementation process, as well as to discuss the lessons learned for the implementation of Algo in occupational therapy.

## 2. Materials and Methods

### 2.1. Design and Variables

This study employed a preobservational-postobservational design (*O*_1_ *X* *O*_2_). In 2009, an initial census (*O*_1_) was conducted to describe the use of skill mix in homecare occupational therapy to meet the needs of elders with bathing difficulties. Then, the integrated KTE intervention was conducted (*X*; independent variable) between 2009 and 2013. At the end of the funding period in 2013, a second census (*O*_2_) was completed to measure changes in clinical practices. Specifically, the dependent variables were the deimplementation of in-house “tools” (*Y*_1_) and the level of utilization of Algo (*Y*_2_), the main expected outcomes by the KTE intervention.

### 2.2. Setting and Participants

The studied population consisted in all the Health and Social Services Centers (HSSCs) of Quebec (Canada). At the time of data collection, the Quebec healthcare system was divided through HSSCs. These HSSCs corresponded to the local services networks (divided according to given territories of the healthcare system) responsible for the access to and continuity of health services [22]. These services were coordinated by a Local Health and Social Services Agency, one for each of the 16 regions of Quebec. They were provided to the population through 95 different HSSCs in 2009 and 94 in 2013 as 1 HSSC had merged with another.

### 2.3. Data Collection

#### 2.3.1. 2009 Census (*O*_1_)

The first census (*O*_1_) took place between May and September 2009. Methods used for both censuses (i.e., phone surveys) followed Dillman and colleagues' recommendations [23]. First, a research assistant contacted the clinical administrators of the Quebec healthcare system homecare services, i.e., in the 95 HSSCs, to solicit their participation. There were no eligibility criteria, since all the HSSCs of the Quebec healthcare system offering homecare services in occupational therapy were involved in this study.

*(1) Data Measurement*. Completed during each appointment, the phone survey initially contained 61 questions and aimed to document different variables to consider in the study of the deimplementation of in-house “tools” (*Y*_1_). The questionnaire was developed based on the professional experience of occupational therapists and researchers involved in the research process. It was pretested with 2 HSSCs and led to minor changes in order to improve clarity (e.g., reformulation). The survey questions allowed to characterize the setting as well as document the use of skill mix and tools related to bathing equipment in homecare occupational therapy with clients having bathing difficulties, for each HSSC. Divided into 3 main sections, the phone survey contains questions, mainly closed-ended, about (1) the structure of services given by the HSSC (e.g., “Is there at least one occupational therapist working in the homecare services of your HSSC?”), (2) the stakeholders involved in the process of recommending bathing equipment (e.g., “In your setting, who recommends the bathing equipment for clients with bathing difficulties?”), and (3) the tools used to this end, if so applicable (e.g., “In your workplace, is there a tool such as a decision tree/observation grid/questionnaire used by non-occupational therapists to guide their choice of assistive devices in the bathroom?”). When interviewees were unable to answer a question, they were invited to refer the interviewer to a colleague.

#### 2.3.2. KTE Intervention: BATH Project (*X*)

The KTE intervention, defined as the BATH project (2009-2013), stands for the French acronym *Besoin d'Aides Techniques lors de l'Hygiène* (i.e., Technical Assistance Needed during Hygiene). It was initiated by a clinician and a homecare occupational therapist working in a HSSC delivering services to urban and rural communities. The team also included two university professors and researchers in clinical gerontology, supervising methodological choices, and two decision-makers, healthcare managers at a Local Health and Social Services Agency. At that time, financial and scientific support was available to allow the clinical occupational therapist to drive the KTE intervention. She had in-depth knowledge of the field and a significant level of peer recognition, factors documented as facilitators for KTE interventions [24].

The operational KTE framework used to guide the KTE intervention (*X*) was structured around five key questions to facilitate knowledge transfer (Table 1): Why?, What?, To whom?, By whom?, and How? [24].

The design of the KTE intervention also relied on both formal and informal ongoing exchanges with stakeholders. The process at the core of the KTE intervention was an iterative dialog between knowledge users and producers to develop, implement, and assess a common tool (i.e., clinical decision algorithm) to replace in-house “tools.” The KTE intervention was comprised of the three steps: (1) development, (2) validation, and (3) assessment of the clinical decision tool.

**Step 1**

*Development*. For the first step, a tool meeting the needs of Quebec HSSCs involving nonoccupational therapists in selecting bathing equipment for homecare clients was developed in collaboration with clinical stakeholders, based on mixed methods. It started with (a) a literature review about skill mix in occupational therapy [30], (b) field observations and interviews with 3 nonoccupational therapists from one HSSC selecting bathing equipment, exploring their needs for support (e.g., In your opinion, what kind of support should be provided to a home health aide involved in selecting bathing equipment?) [31], and (c) a census of in-house “tools” used in the 95 HSSCs [9]. A common tool was then drafted, based on an ongoing iterative process, including those results as well as (d) a synthesis of the 52 in-house “tools” identified, (e) the feedback from 10 clinical occupational therapists working in 10 different HSSCs representing 7 of Quebec's 16 regions, using two questionnaires and one focus group, (f) pretests in a different HSSC, and (g) a translation [11]. The French and English versions of Algo were then ready (September 2011; 4th draft) for steps 2 and 3. Authorization was given to interested HSSCs wanting to implement the 4th draft while Algo was being validated.

Complementary to the development of Algo, 4 knowledge transfer strategies were also created in collaboration with stakeholders in homecare services to support the diffusion and application of the tool during the KTE intervention [31]: (1) the reference manual, (2) the user guide, (3) a website and (4) training offered in the workplace. The reference manual (for occupational therapists) and the user guide (for nonoccupational therapists) were first designed to guide Algo users and other members of the interdisciplinary team. A website was created to promote the tool, answer frequently asked questions, and inform current and potential users. Finally, the nonmandatory training offered in clinical settings allows to present Algo in an interactive way and enhance the stakeholders' understanding of its development and use.

**Step 2**

*Validation*. To achieve the KTE intervention's second step, Algo was validated by comparing the choice of bathing equipment made by nonoccupational therapists using the new tool with the choice made by registered occupational therapists. Eight (8) nonoccupational therapists from 4 HSSCs, 2 of which previously participated to (e) the feedback process during step 1, were trained by the research team to administrate Algo. They used it with 74 elders that requested a bathroom assessment to one of the 4 participating HSSCs [32]. Their bath seat recommendations were compared to those proposed by an occupational therapist (research assistant), which was considered the gold standard.

**Step 3**

*Assessment of the Tool*. For the third step of the KTE intervention, to determine a clinically acceptable threshold of agreement between the recommendations of bathing equipment, a subgroup of 38 elders (out of the 74 above) was assessed a third time by one of the 12 clinical occupational therapists working in one of the same 4 HSSCs that participated to step 2 [32]. After step 3, the 4th draft underwent minor changes (e.g., wording and graphic design) to finalize Algo during the fall of 2012.

In summary, all 16 of Quebec's regions were involved in the KTE intervention, at some point. Prior to the 2013 census (*O*_2_), about 250 people working in 87 Quebec HSSCs collaborated at different times to develop, validate, or test one of the Algo drafts. While 87 HSSCs participated punctually through the development studies (step 1), 4 of them participated in step 2 (validation) and step 3 (assessment of the tool). This description of the KTE intervention illustrates the sequence of steps accomplished during the process, Algo being a component among the KTE intervention results.

#### 2.3.3. 2013 Census (*O*_2_)

**Figure 1:**
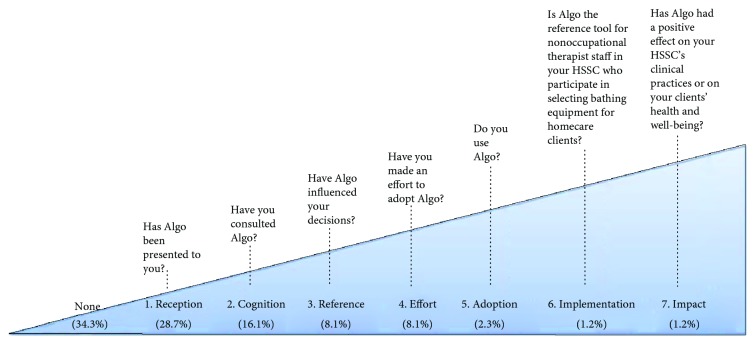
Levels of utilization of Algo (adapted from Knott and Wildavsky, 1980) at the end of the BATH project by Health and Social Services Centers (*n* = 87).

The second census (*O*_2_) was completed between September 2012 and January 2013, using the same methods [23]. A research assistant contacted the clinical administrators of the Quebec healthcare system homecare services a second time to document the same variables. In addition to questions about the in-house “tools” used, questions based on Knott and Wildavsky's seven levels of utilization were also asked during the phone survey in a fourth section (Figure 1) to measure the level of utilization of Algo (*Y*_2_) induced by the KTE intervention (*X*), for a total of 69 questions. Those levels represent a chain of utilization, where one level needs to be reached before moving on to the next [33].

### 2.4. Data Analysis

Descriptive statistics (i.e., frequencies and percentages) were used to summarize the answers to Sections 3 and 4 of the phone survey, respectively, about the use of in-house “tools” in the HSSCs in both censuses (*O*_1_ and *O*_2_), as well as the level of utilization of Algo at the second census (*O*_2_). There is no missing data to address.

### 2.5. Ethical Considerations

The study protocol was conducted in accordance with the Declaration of Helsinki and was approved by the Research Ethics Committee of the Eastern Townships HSSC (MP-CSSS-ESTRIE-09-003). Each interviewee received a thank you letter, and a copy of Algo was mailed to each of them in February of 2013.

## 3. Results

Participation rate in the 2009 census (*O*_1_) was 91% as 86 HSSCs out of a possible 95 answered our questions (3 refused and 6 were not reached). Participation rate in 2013 (*O*_2_) was similar (93%): 87 HSSCs completed the phone survey, 2 refused, and 5 could not be reached. One HSSC participated in neither survey, while 81 participated in both. Interviews lasted 16 minutes on average (range: 5-50 minutes) in 2009, compared to 20 minutes (range: 6-68 minutes) in 2013. A total of 174 individuals were interviewed in 2009 versus 112 in 2013. Interviewees were homecare managers, clinical coordinators, and occupational therapists.

In both censuses (*O*_1_ and *O*_2_), all HSSCs had occupational therapists involved in selecting bathing equipment. In addition, in 2009, 75 HSSCs (87%) resorted to skill mix when advising clients with bathing difficulties, compared to 85 (98%) in 2013. To support decisions by nonoccupational therapists, 58 HSSCs (67%) used in-house “tools” in 2009, in contrast to 71 (81%) in 2013. Of the 81 HSSCs participating in both censuses (*O*_1_ and *O*_2_), 52 used in-house “tools” at both times, 11 HSSCs had started using one in 2013, 4 had stopped using one in 2013, and 14 were using none at either time.

Figure 1 also presents the level of utilization of Algo at the end of the KTE intervention, according to the classification of Knott and Wildavsky [33]. Before Algo was officially available to HSSCs, almost one-third of them (i.e., 25 of the 87 HSSCs) had achieved the first level of utilization, meaning that the prefinal versions of Algo had reached knowledge users. Interviewees reported 6 communication channels by which they were informed about Algo: word of mouth (42%), being involved in data collection for the KTE intervention (23%), publication in a professional journal (16%), communication at a conference (13%), reading in a scientific article (3%), and other (did not remember: 3%).

Fourteen (14) interviewees had moved on to the second level of knowledge utilization, having taken the time to study Algo. Seven (7) were in the process of adopting it: 2 of them were using in-house “tools” in 2013 while 5 had no tools in their setting for nonoccupational therapists. One HSSC, involved in the validation of Algo, did implement it (4th draft) and maintained that the clinical decision algorithm yielded tangible benefits for their clinical practice with clients. Since that particular HSSC was not using any tool in 2009, our results show that no organization had deimplemented their in-house “tool” in favor of Algo during the KTE intervention.

## 4. Discussion

The purpose of this study was to measure the effect of a KTE intervention on the level of utilization of a clinical algorithm (i.e., Algo) to select bathing equipment in occupational therapy. The results from our preobservational-postobservational study revealed that about one-third of knowledge users for which Algo was intended had initiated the utilization process at the end of the KTE intervention. However, none of the participants deimplemented their in-house “tool” to use Algo.

Our hypothesis was that involving end users (i.e., occupational therapists and nonoccupational therapists) within an interactive model of research utilization from the beginning would greatly facilitate the deimplementation of in-house “tools” and their replacement by Algo. Theoretically, collaboration and sustained interactivity between producers and users of knowledge will lead to more applicable research and greater likelihood of its use in practice by considering contextual and individual characteristics [34]. Indeed, developing and testing Algo within HSSCs has therefore probably initiated what Weiss called the “truth test” and the “utility test” [35]. The “truth test” describes the assessment of the research-derived advice on the strength of the underlying evidence, while the “utility test” is the assessment of the advice based on its usefulness to improve practices or solve practical issues. New interventions are often “implemented” as if nothing existed beforehand; however, we contend that “replacement” is much more common than “implementation.” This perspective reinforces the idea that KTE is essentially a competition and selection process [26], where the impact of an innovation will depend on the capacity to replace old ways of doing things. In the case of Algo, even if the KTE intervention was insufficient to induce such a substitution, it may have influenced the utilization of in-house “tools”. For instance, we observed more skill mix in HSSCs for clients with bathing difficulties plus awareness of the upcoming Algo, before abandonment of the in-house “tools.” Knott and Wildavsky's levels [33] illustrate this possibility through intermediate stages of utilization of Algo (e.g., levels 2 to 4) reached by almost one-third of HSSCs in 2013. A longitudinal perspective would be important to adopt since perceived net benefit to patients seems to be a feature influencing both the implementation and deimplementation processes [14]. As this benefit tends to be observed in advanced levels of utilization according to Knott and Wildavsky [33], it appears difficult in a short-term perspective to document the impact of this characteristic on the deimplementation of in-house “tools” in favor of Algo.

Some limitations of this study warrant discussion. First, it does not pinpoint the determining factor(s) for the observed knowledge use by occupational therapists. Moreover, maturation and historical bias could affect internal validity. Indeed, nonmeasured events other than the KTE intervention may have influenced the deimplementation of in-house “tools” and the level of utilization of Algo. Finally, a potential measurement error may have occurred when respondent answers were inaccurate [23]. To minimize this limitation, the data collection method (i.e., phone survey) allowed discussion between interviewers and participants to help them understand the questions. Nevertheless, strengths of this study include its methodological design and high participation rate. All potential participants of the target population were contacted, leading to no sample error. Moreover, an attempt was made to describe the KTE intervention in detail to enhance results generalization, the use of empirical studies about the process of knowledge utilization being scarce, and almost nonexistent, in rehabilitation science [36].

## 5. Conclusions

The study of the deimplementation process in occupational therapy is an emerging field in research. Resulting from a KTE intervention, Algo aims to replace the use of in-house “tools” in homecare services for the selection of selecting bathing equipment in occupational therapy. Despite its mixed success, this KTE intervention provides some interesting insights into the deimplementation process in occupational therapy. It suggests that uptake of knowledge relies on different and interdependent processes. The evidence gathered from close collaboration with numerous stakeholders in the KTE intervention also suggests that elements like interpersonal relations and involvement in the research process, regularly highlighted by the literature, are positively associated with uptake of knowledge. These elements may have as much to do with the de-implementation potential as with the implementation process. A sustainable collaboration between stakeholders during the development of scientifically grounded knowledge such as Algo may initiate the deimplementation of untested practices like in-house “tools,” but seems insufficient to induce a replacement of practices. A longitudinal perspective would be necessary to document the impact of using KTE interventions in the deimplementation process.

## Figures and Tables

**Table 1:** Guidance for the BATH project to enhance knowledge transfer.

Key questions	Description
Why (values)?	(i) Facilitate evidence-based decisions regarding the delivery of homecare services. (ii) Increase access to and quality of services for elders and their caregivers in the context of a shortage of OTs. (iii) Adapt bathrooms to promote the health and safety of caregivers assisting elders during bathing. (iv) Facilitate workers' mobility between HSSCs. (v) Fulfill OTs' professional obligations.
What?	(i) Target a common tool for non-OTs involved in selecting bathing equipment in HSSCs. (ii) Change clinician behavior, which will be easier if the common tool presents clear actions rather than ideas and concepts [24, 25].
To whom?	(i) Focus on homecare OTs (*n* ≈ 800) because individual interventions have more effect than collective ones [26] and because dissemination needs to be targeted to a specific audience [27]. (ii) Collaborate with homecare managers since context has a major influence on clinicians' behavior [24].
By whom?	(i) Messengers should be selected according to the target audience and could vary from one knowledge transfer strategy to another: the person or group of persons should be credible, influential, and have strong communication skills and leadership [24].
How?	(i) Use interactive models of research utilization to develop knowledge and provide solutions [19]. (ii) Use multiple knowledge transfer strategies, which is more effective than a single strategy [28], within budget limits. (iii) Focus on tactical logic to facilitate decisions [29], contrasting an evidence-based common tool to in-house “tools” casting doubt on the quality of services and questioning OTs' professional liability.
Why (objectives)?	(i) Deimplement in-house “tools” and utilize Algo for non-OTs involved in selecting bathing equipment in HSSCs.

OT: occupational therapist; HSSC: Health and Social Services Center.

## Data Availability

The quantitative data used to support the findings of this study are available from the corresponding author upon request.
